# Diagnostic Accuracy of T-SPOT.TB Assay for Tuberculous Meningitis: An Updated Meta-Analysis

**DOI:** 10.3389/fneur.2020.00866

**Published:** 2020-09-03

**Authors:** Ying Luo, Ying Xue, Xueyun Guo, Qun Lin, Liyan Mao, Guoxing Tang, Huijuan Song, Feng Wang, Ziyong Sun

**Affiliations:** ^1^Department of Laboratory Medicine, Tongji Medical College, Tongji Hospital, Huazhong University of Science and Technology, Wuhan, China; ^2^Department of Clinical Immunology, Tongji Medical College, Tongji Hospital, Huazhong University of Sciences and Technology, Wuhan, China; ^3^Department of Dermatology, Tongji Medical College, Tongji Hospital, Huazhong University of Science and Technology, Wuhan, China

**Keywords:** tuberculous meningitis, T-SPOT.TB, peripheral blood, cerebrospinal fluid, diagnosis, meta-analysis

## Abstract

**Background:** The role of T-SPOT.TB (T-SPOT) assay for tuberculous meningitis (TBM) diagnosis has not been fully assessed. Here, we conducted an updated meta-analysis to evaluate the diagnostic accuracy of peripheral blood (PB) T-SPOT and cerebrospinal fluid (CSF) T-SPOT for diagnosing TBM.

**Methods:** Relevant studies in the PubMed database, EmBase database, Cochrane database, Scopus database, Google Scholar, China National Knowledge Internet, and Wan-Fang database were retrieved from August 1, 2005, to June 22, 2020. Statistical analysis was performed using Stata, Revman, and Meta-Disc software. The pooled sensitivity, specificity, positive likelihood ratio (PLR), negative likelihood ratio (NLR), diagnostic odds ratio (DOR), summary receiver operating characteristic curves, and the area under the curve were determined and analyzed.

**Results:** A total of 27 studies were eligible for inclusion within the meta-analysis. The pooled sensitivity and specificity of PB T-SPOT for TBM diagnosis were 0.78 (95% CI, 0.76–0.81) and 0.68 (95% CI, 0.66–0.71), respectively, whereas the pooled PLR, NLR, and DOR were 2.80 (95% CI, 2.29–3.42), 0.32 (95% CI, 0.27–0.38), and 10.08 (95% CI, 7.21–14.08), respectively. On the other hand, the pooled sensitivity and specificity of CSF T-SPOT on diagnosing TBM were 0.76 (95% CI, 0.72–0.80) and 0.88 (95% CI, 0.85–0.90), respectively, whereas the pooled PLR, NLR, and DOR were 5.92 (95% CI, 4.25–8.25), 0.28 (95% CI, 0.21–0.39), and 29.05 (95% CI, 16.40–51.45), respectively. The area under the summary receiver operating characteristic curve values of PB T-SPOT and CSF T-SPOT for TBM diagnosis were 0.83 (95% CI, 0.80–0.86) and 0.92 (95% CI, 0.89–0.94), respectively.

**Conclusions:** CSF T-SPOT showed a higher specificity compared with PB T-SPOT for diagnosing TBM. Both two T-SPOT assays have considerable potential in improving the diagnosis of TBM. Furthermore, the standardization of the operating procedure is further needed when performing CSF T-SPOT.

## Introduction

Tuberculosis (TB) remains a global health problem, with an estimated 10.0 million new cases and 1.5 million deaths resulting from the disease in 2018 (1). Extrapulmonary tuberculosis (EPTB) represents about 15% of all TB cases recognized by the World Health Organization in 2018 (1). Tuberculous meningitis (TBM) is the most lethal and disabling form of EPTB. It was reported that TBM accounted for 2.9–6.8% of extrapulmonary presentations (2–4). The global burden of TBM is estimated to be more than 100,000 new cases per year (5). Rapid and accurate diagnosis of TBM is of paramount importance in reducing morbidity and mortality. Therefore, innovative tools with higher diagnostic efficiency are indispensable for the control and management of TBM.

The use of smear microscopy in cerebrospinal fluid (CSF) remains consistently difficult owing to the low numbers of bacilli present in this paucibacillary disease (6). Mycobacterial culture displays an increased sensitivity but is limited by a long turnaround time, which results in delayed clinical decision-making (7). Molecular technologies such as GeneXpert MTB/RIF (manufactured by Cepheid) indeed improve the diagnostic value for TB infection, but these methods still show a limited value according to the paucibacillary nature of the disease (8). In total, the present conventional microbiological methods provide restricted efficiency in TBM diagnosis due to the unsatisfactory sensitivity and are time-consuming and costly (9). For biochemical indicators, the levels of adenosine deaminase (ADA) and interferon-gamma in CSF also display potential diagnostic value, but the performance of these indicators is also not satisfactory (10, 11). Besides, brain imaging has long been part of the diagnostic evaluation of TBM (12). However, few studies have defined the diagnostic performance of the potential features, and the appearance of imaging modalities would be influenced by the status, including age and underlying infection (13). Apart from these technologies, clinical scoring systems have also been introduced to apply in this area (14, 15). The major limitation of these diagnostic rules is that their utility is variable in different settings, and few of these scoring systems have been externally validated. The findings vary according to the population origin, age, and HIV status accounting for much of this variation (15). Also, some emerging approaches, including GeneXpert MTB/RIF Ultra (16, 17), droplet digital PCR (18), and exosome DNA (19), have been developed aiming to address the issue recently, but few have been subject to enough repeated validation.

T-SPOT.TB (T-SPOT), one of two commercially interferon-gamma release assays, has also been developed to diagnose TB infection (20). It was reported that both peripheral blood (PB) and CSF could be used to perform T-SPOT and that PB T-SPOT and CSF T-SPOT showed different diagnostic accuracy in TBM (21). Although a previous meta-analysis was conducted based on studies before 2015 (21), several new pieces of research were noted in recent years. Therefore, this study aims to evaluate the diagnostic accuracy of PB T-SPOT and CSF T-SPOT in TBM and comprehensively compare the efficiency between T-SPOT and other existing methods.

## Materials and Methods

This meta-analysis was conducted following the guidelines of the Preferred Reporting Items for Systematic Reviews and Meta-Analyses statement (22). Given that the present study was a meta-analysis of published researches, the approval from the institutional ethics committee and patient consent were not available.

### Search Strategy

All relevant individual studies performed were searched on human subjects published from August 1, 2005, to June 22, 2020, in PubMed database, EmBase database, Cochrane database, Scopus database, Google Scholar, China National Knowledge Internet, and Wan-Fang database. The search terms were used as the follows: (“tuberculous” or “tuberculosis” or “tubercular” or “TB” or “mycobacterium” or “mycobacterial”) and (“meningeal” or “meningitis” or “meningitides”) and (“enzyme-linked immunospot” or “T-SPOT” or “T-SPOT.TB” or “ELISpot” or “interferon-gamma release assays” or “interferon-gamma assays” or “IGRA” or “interferon” or “interferon-gamma” or “gamma-interferon” or “IFN” or “T cell-based assay” or “T cell assays”). Relevant articles related to the keywords were included.

### Study Selection Criteria

To be included in the meta-analysis, studies had to meet the following criteria: (1) original data on the evaluation of diagnostic value; (2) a case–control design and clear diagnosis; and (3) sufficient parameters including at least specificity and sensitivity, together with the corresponding 95% confidence interval (CI), and the number of the included patients. Ying Luo and Ying Xue independently reviewed and assessed study eligibility, and disagreements were resolved by a third author (Ziyong Sun).

### Data Extraction and Quality Assessment

Two investigators (Ying Luo and Ying Xue) independently extracted the following information from each study: first author, country, year of publication, study design, numbers of participants, sensitivity, specificity, and values of true-positive, false-positive, true-negative, and false-negative. The methodological quality of the studies included was assessed using the criteria of the Quality Assessment of Diagnostic Accuracy Studies-2 (23).

### Statistical Analysis

The analysis was performed using Stata (version 14; Stata Corporation, TX, USA), Revman (version 5.3; The Nordic Cochrane Center, The Cochrane Collaboration, Copenhagen, Denmark), and Meta-Disc (version 1.4; XI Cochrane Colloquium, Barcelona, Spain). Data from individual studies were pooled using a random-effect model and used to generate values for the following measures of test accuracy: sensitivity, specificity, positive likelihood ratio (PLR), negative likelihood ratio (NLR), diagnostic odds ratio (DOR) with corresponding 95% CI, and a summary receiver operating characteristic (SROC) curve, which was made to present the individual assessment of sensitivity and specificity for each study. Heterogeneity was assessed by the *I*^2^ statistic (24), and publication bias was evaluated using Deeks' funnel plot (25).

## Results

### Characteristics of the Included Studies

**Figure 1:**
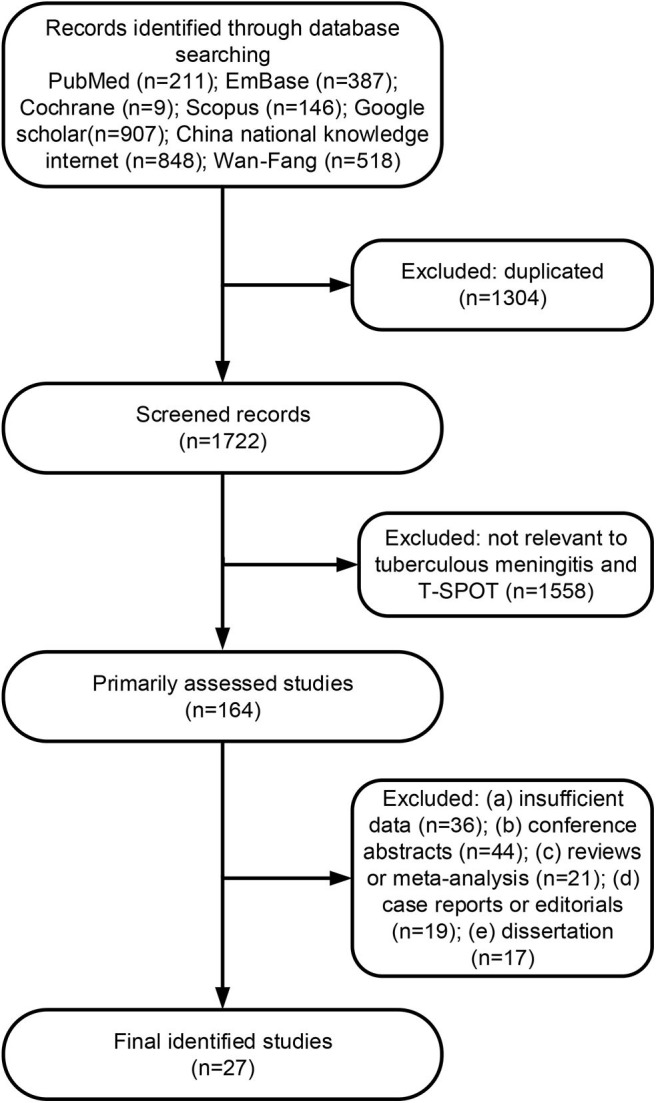
Flowchart diagram for study selection and inclusion.

A total of 3,026 citations were found for patients with TBM diagnosed by T-SPOT (Figure 1). After independent reviews, 27 studies met the inclusion criteria and were finally included into meta-analysis (Table 1 and Figure 1). The PB-based T-SPOT was performed in 25 studies (26–35, 37–42, 44–52), and the CSF-based T-SPOT was used in 16 studies (26–29, 31, 32, 36–39, 41, 43, 46, 48, 50, 51). Head-to-head comparisons of the diagnostic accuracies of PB T-SPOT against CSF T-SPOT were found in 14 studies. The characteristics of the 27 eligible studies, such as country, TB burden, and patient number, are presented in Table 1. The patients of these studies came from six countries and three continents. Of the 27 articles, 21 studies reported data from areas with high TB burden. The amount of cerebrospinal fluid mononuclear cells (CSFMCs) added per well in 11 studies was 2.5 × 10^5^, and the amount of CSFMCs' in the remaining five studies was lower than 2.5 × 10^5^ (Table 1). The total sample size in studies performed on PB was 2,312, comprising 989 patients with TBM and 1,323 non-TBM controls, and sample size in studies based on CSF was 1,252, comprising 538 patients with TBM and 714 non-TBM controls.

**Table 1:** Summary characteristics of studies included in meta-analysis.

**Study number**	**References**	**Country**	**Continent**	TB burden^*^	**Study design**	**Samples**	**TBM/non-TBM patients recruited**	**PBMCs or CSFMCs/well**	**Test results**			
									**TP**	**FP**	**FN**	**TN**
1	Kim et al. (26)	Korea	Asia	Intermediate	Prospective	Peripheral blood	11/24	2.5 × 10^5^	10	9	1	15
						Cerebrospinal fluid	4/12	2.5 × 10^5^	3	3	1	9
2	Thomas et al. (27)	India, UK, Germany	Asia and Europe	High^†^	Prospective	Peripheral blood	11/8	2.5 × 10^5^	9	2	2	6
						Cerebrospinal fluid	10/7	2.5 × 10^5^	9	0	1	7
3	Kim et al. (28)	Korea	Asia	Intermediate	Prospective	Peripheral blood	31/53	2.5 × 10^5^	22	23	9	30
						Cerebrospinal fluid	22/28	2.5 × 10^5^	13	3	9	25
4	Patel et al. (29)	South Africa	Africa	High	Prospective	Peripheral blood	37/50	2.5 × 10^5^	33	33	4	17
						Cerebrospinal fluid	38/48	3 × 10^4^ -2.5 × 10^5^	32	13	6	35
5	Cho et al. (30)	Korea	Asia	Intermediate	Prospective	Peripheral blood	35/87	2.5 × 10^5^	26	47	9	40
6	Park et al. (31)	Korea	Asia	Intermediate	Prospective	Peripheral blood	25/57	2.5 × 10^5^	22	24	3	33
						Cerebrospinal fluid	25/57	2.5 × 10^5^	18	12	7	45
7	Zhang et al. (32)	China	Asia	High	Retrospective	Peripheral blood	30/30	2.5 × 10^5^	23	4	7	26
						Cerebrospinal fluid	30/30	1 × 10^4^	28	1	2	29
8	Chen et al. (33)	China	Asia	High	Retrospective	Peripheral blood	40/18	2.5 × 10^5^	27	2	13	16
9	Lu et al. (34)	China	Asia	High	Retrospective	Peripheral blood	30/39	2.5 × 10^5^	21	5	9	34
10	Lv et al. (35)	China	Asia	High	Retrospective	Peripheral blood	32/27	2.5 × 10^5^	23	4	9	23
11	Mou et al. (36)	China	Asia	High	Retrospective	Cerebrospinal fluid	47/45	2.5 × 10^5^	38	5	9	40
12	Qin et al. (37)	China	Asia	High	Prospective	Peripheral blood	12/28	2.5 × 10^5^	10	5	2	23
						Cerebrospinal fluid	12/28	≤2.5 × 10^5^	11	2	1	26
13	Cui et al. (38)	China	Asia	High	Retrospective	Peripheral blood	40/66	2.5 × 10^5^	29	9	11	57
						Cerebrospinal fluid	40/66	1 × 10^4^	38	5	2	61
14	Li et al. (39)	China	Asia	High	Retrospective	Peripheral blood	53/36	2.5 × 10^5^	48	9	5	27
						Cerebrospinal fluid	53/36	2.5 × 10^5^	38	3	15	33
15	Lu et al. (40)	China	Asia	High	Retrospective	Peripheral blood	30/39	2.5 × 10^5^	21	5	9	34
16	Park et al. (41)	Korea	Asia	Intermediate	Prospective	Peripheral blood	46/159	2.5 × 10^5^	38	66	8	93
						Cerebrospinal fluid	38/109	2.5 × 10^5^	28	16	10	93
17	Wang et al. (42)	China	Asia	High	Retrospective	Peripheral blood	54/34	2.5 × 10^5^	45	10	9	24
18	Li et al. (43)	China	Asia	High	Retrospective	Cerebrospinal fluid	52/44	2.5 × 10^5^	51	10	1	34
19	Lu et al. (44)	China	Asia	High	Retrospective	Peripheral blood	61/85	2.5 × 10^5^	38	23	23	62
20	Ma et al. (45)	China	Asia	High	Retrospective	Peripheral blood	74/80	2.5 × 10^5^	56	33	18	47
21	Pan et al. (46)	China	Asia	High	Prospective	Peripheral blood	53/37	2.5 × 10^5^	48	9	5	28
						Cerebrospinal fluid	51/36	1 × 10^5^-2.5 × 10^5^	31	1	20	35
22	Wang et al. (47)	China	Asia	High	Retrospective	Peripheral blood	29/36	2.5 × 10^5^	26	9	3	27
23	Song et al. (48)	China	Asia	High	Retrospective	Peripheral blood	76/57	2.5 × 10^5^	59	9	17	48
						Cerebrospinal fluid	76/57	2.5 × 10^5^	48	1	28	56
24	Wu et al. (49)	China	Asia	High	Retrospective	Peripheral blood	55/76	2.5 × 10^5^	45	18	10	58
25	Yang et al. (50)	China	Asia	High	Retrospective	Peripheral blood	30/66	2.5 × 10^5^	28	15	2	51
						Cerebrospinal fluid	30/66	2.5 × 10^5^	20	8	10	58
26	Kwon et al. (51)	Korea	Asia	Intermediate	Prospective	Peripheral blood	10/45	2.5 × 10^5^	5	10	5	35
						Cerebrospinal fluid	10/45	2.5 × 10^5^	3	4	7	41
27	Xu et al. (52)	China	Asia	High	Retrospective	Peripheral blood	84/86	2.5 × 10^5^	63	35	21	51

* *Refer to Global Tuberculosis Report 2019; Most participants were enrolled in India. TB, tuberculosis; TBM, tuberculous meningitis; PBMCs, peripheral blood mononuclear cells; CSFMCs, cerebrospinal fluid mononuclear cells; TP, true positive; FP, false positive; FN, false negative; TN, true negative*.

### Quality Assessment

**Figure 2:**
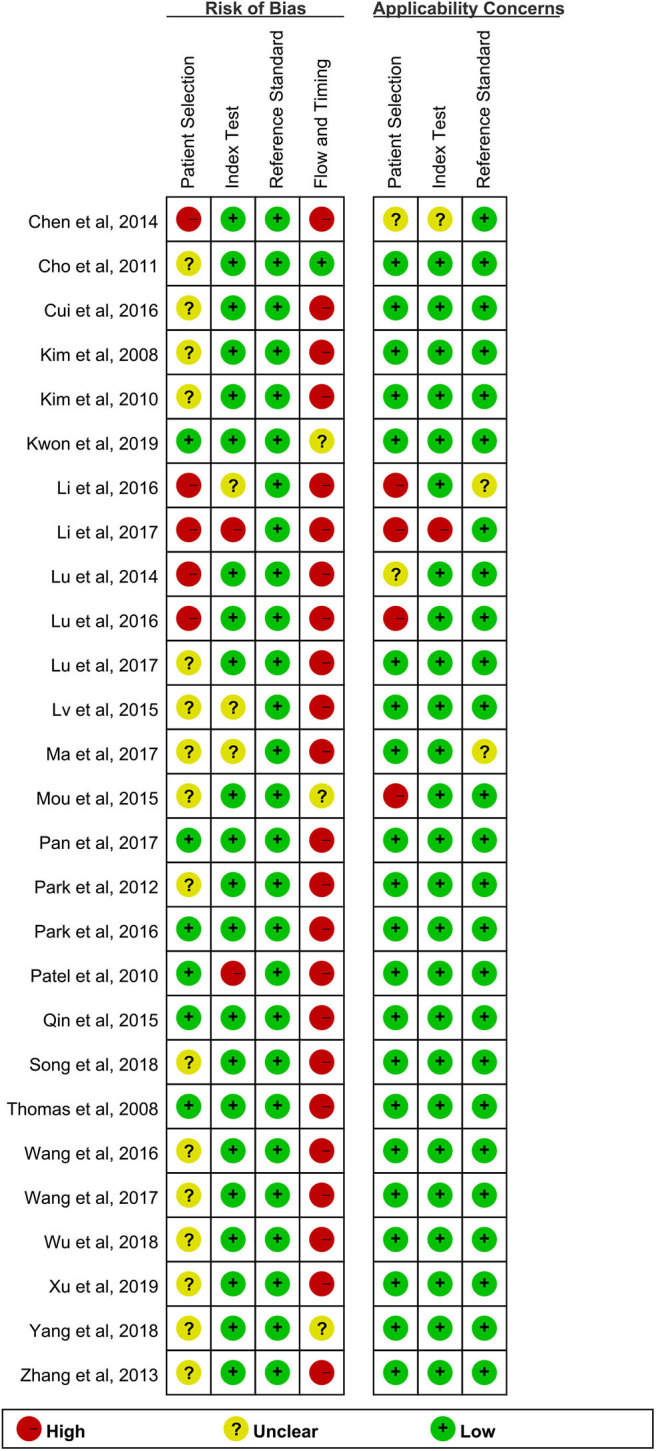
Quality evaluation of articles included.

All studies provided detailed diagnostic standards used to define TBM. There was no low risk of bias in all studies (Figure 2). The risk of bias for the index test domain largely resulted from a lack of information on blinding.

### Pooled Diagnostic Accuracy in Peripheral Blood and Cerebrospinal Fluid T-SPOT

**Figure 3:**
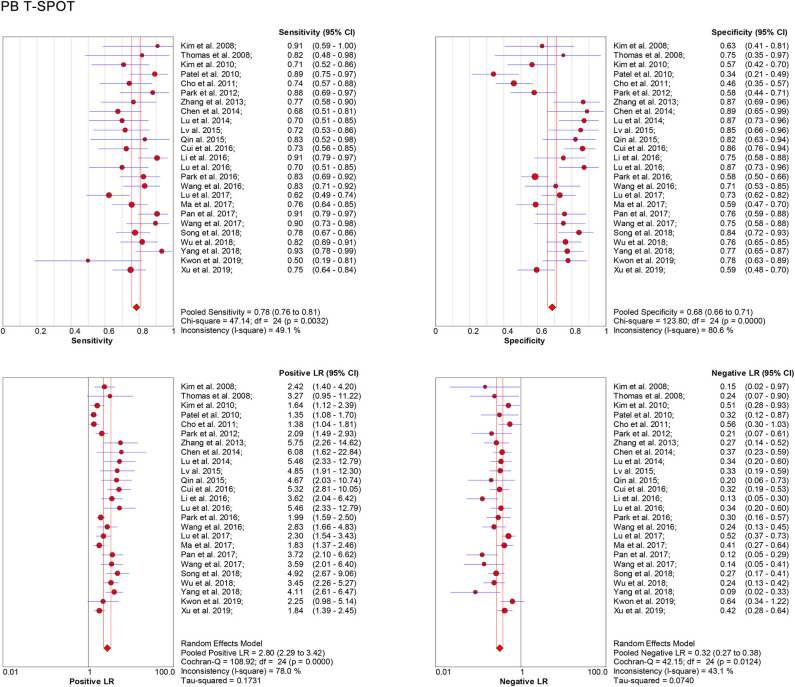
Forest plot showing estimates of sensitivity, specificity, PLR, and NLR for PB T-SPOT for the diagnosis of TBM. Solid dots represent the point estimates of sensitivity, specificity, PLR, and NLR from each study. Size of solid dots reflects the total number of cases and controls. Error bars indicate 95% CI. Pooled results are shown as diamonds. PLR, positive likelihood ratio; NLR, negative likelihood ratio; PB, peripheral blood; TBM, tuberculous meningitis; CI, confidence interval.

Data from 25 studies based on PB T-SPOT were subjected to a meta-analysis to generate pooled values for diagnostic accuracy parameters, as follows: the sensitivities varied from 0.50 to 0.93, with a pooled estimate of 0.78 (95% CI, 0.76–0.81). The specificities varied from 0.34 to 0.87, with a pooled estimate of 0.68 (95% CI, 0.66–0.71) (Figure 3). The pooled estimates for PLR, NLR, and DOR were 2.80 (95% CI, 2.29–3.42), 0.32 (95% CI, 0.27–0.38), and 10.08 (95% CI, 7.21–14.08), respectively (Figures 3, **5A**), and area under the SROC curve, 0.83 (95% CI, 0.80–0.86) (**Figure 6A**). Data from the studies showed various heterogeneity for these accuracy parameters, based on *I*^2^ values of 49.1% for sensitivity, 80.6% for specificity, 78.0% for PLR, 43.1% for NLR, and 56.1% for DOR (Figures 3, **5A**).

**Figure 4:**
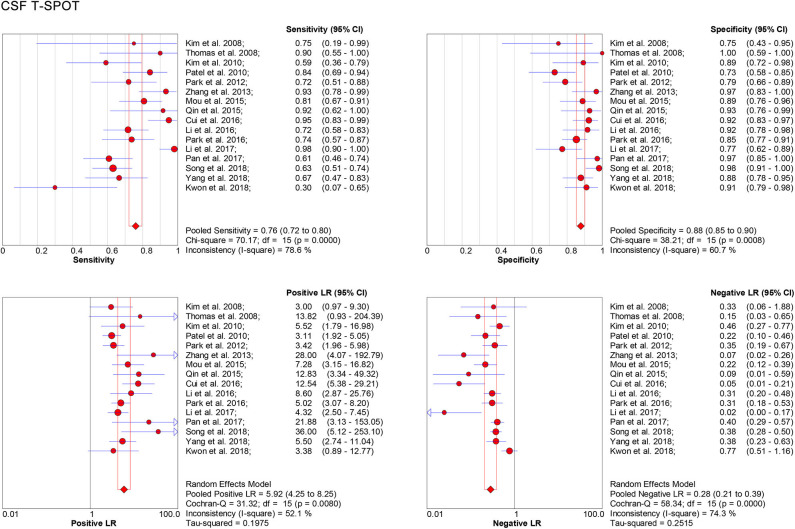
Forest plot showing estimates of sensitivity, specificity, PLR, and NLR for CSF T-SPOT for the diagnosis of TBM. Solid dots represent the point estimates of sensitivity, specificity, PLR, and NLR from each study. Size of solid dots reflects the total number of cases and controls. Error bars indicate 95% CI. Pooled results are shown as diamonds. PLR, positive likelihood ratio; NLR, negative likelihood ratio; CSF, cerebrospinal fluid; TBM, tuberculous meningitis; CI, confidence interval.

**Figure 5:**
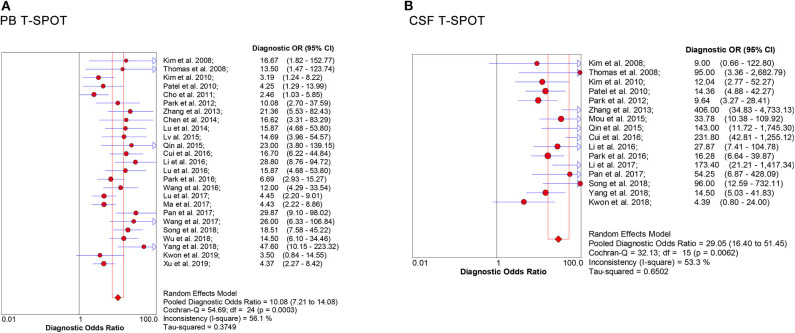
Forest plot of estimates of DOR for **(A)** PB T-SPOT and **(B)** CSF T-SPOT for the diagnosis of TBM. Point estimates of DOR from each study are shown as solid dots whose size reflects the total number of cases and controls. Error bars show 95% CI. DOR, diagnostic odds ratio; PB, peripheral blood; CSF, cerebrospinal fluid; TBM, tuberculous meningitis.

**Figure 6:**
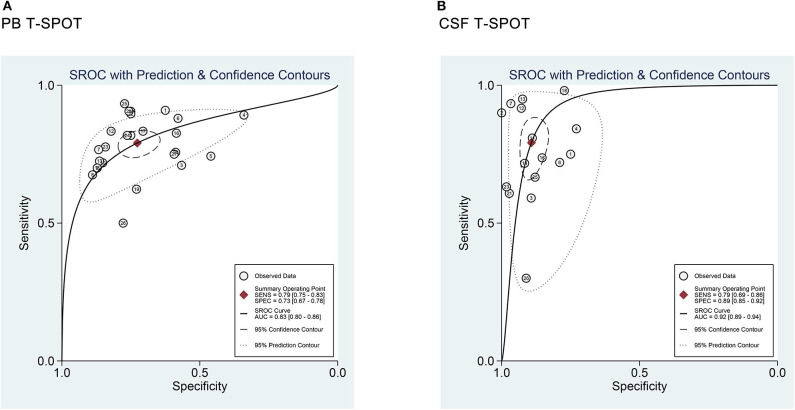
SROC curves for T-SPOT in the **(A)** PB and **(B)** CSF for TBM diagnosis. Each study included in the meta-analysis is shown as a solid dot. Numbers indicate the included numbers of studies. Regression SROC curves summarize the overall diagnostic accuracy. PB, peripheral blood; CSF, cerebrospinal fluid; TBM, tuberculous meningitis; SROC, summary receiver operating characteristic.

One the other hand, data based on 16 studies showed the pooled sensitivity and specificity for CSF T-SPOT were 0.76 (95% CI, 0.72–0.80) and 0.88 (95% CI, 0.85–0.90), respectively (Figure 4). The PLR, NLR and DOR of CSF T-SPOT in TBM diagnosis were 5.92 (95% CI, 4.25–8.25), 0.28 (95% CI, 0.21–0.39), and 29.05 (95% CI, 16.40–51.45) (Figures 4, 5B), respectively. The area under the SROC curve for CSF T–SPOT on diagnosing TBM was 0.92 (95% CI, 0.89–0.94) (Figure 6B). Data from the studies showed various heterogeneity for these accuracy indexes, based on *I*^2^ values of 78.6% for sensitivity, 60.7% for specificity, 52.1% for PLR, 74.3% for NLR, and 53.3% for DOR (Figures 4, 5B).

In total, the pooled sensitivity of PB T-SPOT for TBM diagnosis was slightly higher than that of CSF T-SPOT (0.78 vs. 0.76). However, the pooled specificity of PB T-SPOT was obviously lower than that of CSF T-SPOT (0.68 vs. 0.88).

### Multiple Regression Analysis

The regression analysis was performed for CSF T-SPOT on heterogeneous sources. It was found that experimental design, TB burden, number of patients, and CSFMCs added to per well did not significantly affect the diagnostic utility of CSF T-SPOT for TBM (Tables 2, 3).

**Table 2:** Subgroup analyses for exploration of factors influencing heterogeneity in CSF T-SPOT assay.

**Variables**	**Category (number of studies)**	**Pooled sensitivity (95% CI)**	***I*^**2**^**	**Pooled specificity (95% CI)**	***I*^**2**^**	**Pooled diagnostic odds ratio (95% CI)**
Design	Prospective (9)	0.70 (0.64–0.77)	60.1%	0.85 (0.81–0.89)	57.4%	14.88 (8.86–24.99)
	Retrospective (7)	0.80 (0.75–0.84)	86.5%	0.90 (0.87–0.93)	60.6%	61.29 (24.53–153.10)
TB burden	High TB prevalence (11)	0.78 (0.74–0.82)	82.1%	0.89 (0.86–0.92)	67.6%	51.27 (25.47–103.20)
	Intermediate TB prevalence (5)	0.66 (0.55–0.75)	46.2%	0.85 (0.80–0.89)	5.9%	11.25 (6.35–19.93)
Sample	>60 (10)	0.76 (0.72–0.80)	81.3%	0.87 (0.84–0.90)	70.2%	29.20 (15.81–53.93)
	≤60 (6)	0.76 (0.66–0.85)	77.4%	0.91 (0.86–0.95)	12.1%	30.29 (7.03–130.54)
CSFMCs/well	2.5 × 10^5^ (11)	0.73 (0.68–0.78)	76.4%	0.87 (0.84–0.90)	52.1%	19.51 (11.39–33.44)
	≤2.5 × 10^5^ (5)	0.82 (0.75–0.87)	82.6%	0.89 (0.84–0.93)	75.9%	84.63 (20.43–350.66)

*CSF, cerebrospinal fluid; CSFMCs, cerebrospinal fluid mononuclear cells; TB, tuberculosis; CI, confidence interval*.

**Table 3:** Weighted meta-regression to assess the effects of various factors on the diagnostic accuracy of CSF T-SPOT assay.

**Covariate**	**Coefficient**	**RDOR (95% CI)**	***P***
Design			
Prospective (9)	−1.528	0.22 (0.03–1.51)	0.11
Retrospective (7)			
TB burden			
High TB prevalence (11)	−0.045	0.96 (0.10–9.06)	0.97
Intermediate TB prevalence (5)			
Sample			
>60 (10)	−0.537	0.58 (0.14–2.38)	0.41
≤60 (6)			
CSFMCs/well			
2.5 × 10^5^ (11)	−1.302	0.27 (0.04–1.75)	0.15
≤2.5 × 10^5^ (5)			

*CSF, cerebrospinal fluid; RDOR, relative diagnostic odds ratio; TB, tuberculosis; CSFMCs, cerebrospinal fluid mononuclear cells; CI, confidence interval*.

### Publication Bias

**Figure 7:**
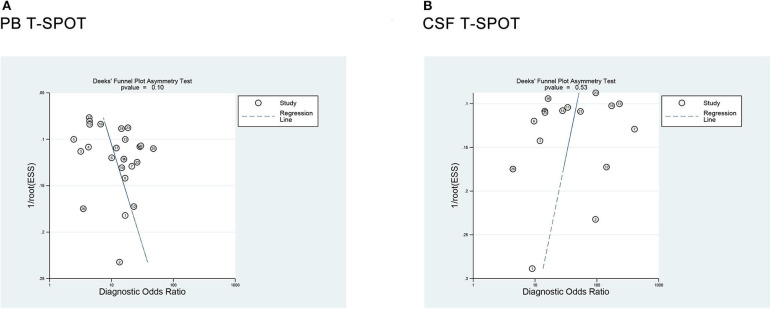
Deeks' funnel plot asymmetry test for the evaluation of potential publication bias in **(A)** PB T-SPOT and **(B)** CSF T-SPOT studies. PB, peripheral blood; CSF, cerebrospinal fluid.

Deeks' funnel plot asymmetry test was used to assess the publication bias. The *P*-values were 0.10 for PB T-SPOT and 0.53 for CSF T-SPOT, which indicated a low risk for publication bias among all included studies (Figure 7).

### Diagnostic Value of T-SPOT for Tuberculous Meningitis When Comparing Microbiologically Confirmed Tuberculous Meningitis With Non-tuberculous Meningitis

**Figure 8:**
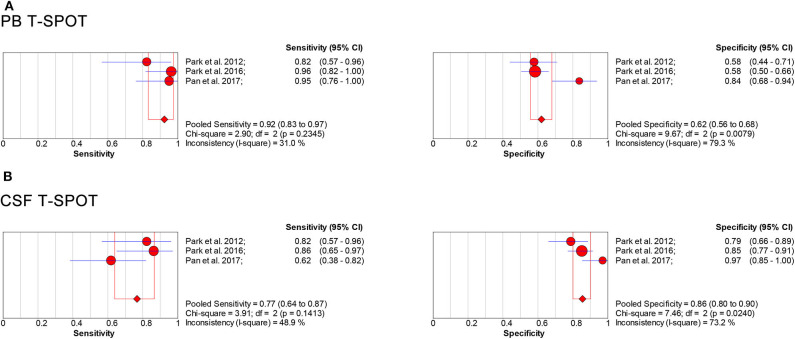
Forest plot showing estimates of sensitivity and specificity for PB T-SPOT **(A)** and CSF T-SPOT **(B)** for the diagnosis of TBM when comparing microbiologically confirmed TBM with non-TBM. Solid dots represent the point estimates of sensitivity and specificity from each study. Size of solid dots reflects the total number of cases and controls. Error bars indicate 95% CI. Pooled results are shown as diamonds. PB, peripheral blood; CSF, cerebrospinal fluid; TBM, tuberculous meningitis; CI, confidence interval.

We analyzed the included literature and identified the T-SPOT results in patients with microbiologically confirmed TBM and non-TBM in three studies (Table 4) (31, 41, 46). The total sample size in studies performed on PB was 319, comprising 66 patients with confirmed TBM and 253 non-TBM; the sample size in studies based on CSF was 262, comprising 60 patients with confirmed TBM and 202 non-TBM. The data showed the pooled sensitivity and specificity of PB T-SPOT were 0.92 (95% CI, 0.83–0.97) and 0.62 (95% CI, 0.56–0.68), respectively (Figure 8A). Besides, the pooled sensitivity and specificity of CSF T-SPOT were 0.77 (95% CI, 0.64–0.87) and 0.86 (95% CI, 0.80–0.90), respectively (Figure 8B).

**Table 4:** Summary characteristics of studies comparing confirmed TBM with non-TBM.

**References**	**Country**	**Continent**	TB burden^*^	**Study design**	**Samples**	**TBM/non-TBM patients recruited**	**PBMCs or CSFMCs/well**	**Test results**			
								**TP**	**FP**	**FN**	**TN**
Park et al. (31)	Korea	Asia	Intermediate	Prospective	Peripheral blood	17/57	2.5 × 10^5^	14	24	3	33
					Cerebrospinal fluid	17/57	2.5 × 10^5^	14	12	3	45
Park et al. (41)	Korea	Asia	Intermediate	Prospective	Peripheral blood	28/159	2.5 × 10^5^	27	66	1	93
					Cerebrospinal fluid	22/109	2.5 × 10^5^	19	16	3	93
Pan et al. (46)	China	Asia	High	Prospective	Peripheral blood	21/37	2.5 × 10^5^	20	6	1	31
					Cerebrospinal fluid	21/36	1 × 10^5^ -2.5 × 10^5^	13	1	8	35

* *Refer to Global Tuberculosis Report 2019; TB, tuberculosis; TBM, tuberculous meningitis; PBMCs, peripheral blood mononuclear cells; CSFMCs, cerebrospinal fluid mononuclear cells; TP, true positive; FP, false positive; FN, false negative; TN, true negative*.

### Comparison of the Diagnostic Accuracy of Various Approaches for Tuberculous Meningitis

In this study, we also compared the diagnostic value of T-SPOT with other tests from meta-analyses. GeneXpert MTB/RIF was reported to have higher specificity than both PB and CSF T-SPOT for diagnosing TBM. However, this molecular tool was not sensitive enough to rule out non-TBM cases (53, 54). GeneXpert MTB/RIF Ultra, the next generation of GeneXpert MTB/RIF, showed a slightly higher sensitivity than both PB and CSF T-SPOT (55). Meanwhile, the specificity of this new technology was high. Besides, the overall accuracy of ADA was superior to PB T-SPOT but comparable with CSF T-SPOT (11, 56, 57) (Table 5).

**Table 5:** Meta-analyses assessing the performance of various methods for diagnosing TBM.

**Methods**	**References**	**TBM/non-TBM patients**	**Included studies**	**AUC (95% CI)**	**Sensitivity (95% CI)**	**Specificity (95% CI)**	**PLR (95% CI)**	**NLR (95% CI)**	**DOR (95% CI)**
GeneXpert MTB/RIF	Kohli et al. (54)	433/3,341	29	NA	0.71 (0.60–0.80)	0.98 (0.97–0.99)	NA	NA	NA
	Chen et al. (53)	884/3,211	14	0.76 (NA)	0.63 (0.59–0.66)	0.98 (0.97–0.99)	20.9 (12.7–52.8)	0.40 (0.32–0.50)	71.49 (32.64–156.56)
GeneXpert MTB/RIF Ultra	Donovan et al. (55)	128/321	4	NA	0.81 (0.73–0.88)	1.00 (0.99–1.00)	NA	NA	NA
ADA	Xu et al. (56)	356/1,083	10	0.92 (NA)	0.79 (0.75–0.83)	0.91 (0.89–0.93)	6.85 (4.11–11.41)	0.29 (0.19–0.44)	26.93 (12.73–56.97)
	Tuon et al. (57)	380/712	13	0.91 (NA)	0.74 (0.69–0.79)	0.87 (0.84–0.89)	5.61 (3.10–10.30)	0.30 (0.18–0.47)	24.22 (9.23–63.64)
	Pormohammad et al. (11)	741/1,169	20	0.96 (NA)	0.89 (0.84–0.92)	0.91 (0.87–0.93)	9.4 (7–12.8)	0.12 (0.09–0.17)	77 (45–132)
PB T-SPOT	Luo et al., this study	989/1,323	25	0.83 (0.80–0.86)	0.78 (0.76–0.81)	0.68 (0.66–0.71)	2.80 (2.29–3.42)	0.32 (0.27–0.38)	10.08 (7.21–14.08)
CSF T-SPOT	Luo et al., this study	538/714	16	0.92 (0.89–0.94)	0.76 (0.72–0.80)	0.88 (0.85–0.90)	5.92 (4.25–8.25)	0.28 (0.21–0.39)	29.05 (16.40–51.45)

*ADA, adenosine deaminase; PB, peripheral blood; CSF, cerebrospinal fluid; TBM, tuberculous meningitis; AUC, area under the curve; PLR, positive likelihood ratio; NLR, negative likelihood ratio; DOR, diagnostic odds ratio; CI, confidence interval; NA, not applicable*.

## Discussion

The central nervous system disease caused by *Mycobacterium tuberculosis* (MTB) is highly devastating (58). TBM is the most common form of central nervous system TB (59). Appropriate diagnosis and treatment are paramount tasks to control and end TB (60, 61). Delayed diagnosis is associated with a poor prognosis in TBM, and improved diagnosis has the potential to improve national and international surveillance of numbers of cases and trends in incidence. However, the development in this area has been sluggish in recent years. Some progress has been made but has not yet translated into outcome benefits in clinical practice (62). Further investigations should always be sought. The diagnosis of TBM remains difficult to ascertain due to no specific clinical features and the poor efficiency of the current assays, so the disease might be underreported. Thus, rapid, sensitive, and affordable diagnostic tests that can be used at the point of care are crucially needed, particularly in the regions with a high TB burden.

Our study demonstrated that CSF T-SPOT is slightly less sensitive but obviously more specific than PB T-SPOT for diagnosing TBM, which indicated that CSF T-SPOT could assist in TBM diagnosis. The overall efficiency of CSF T-SPOT shows some but not an obvious advantage over that of PB T-SPOT, which is consistent with the conclusion of a previous meta-analysis (21). These results support that TB-specific cells would accumulate in infection sites in TB (63). However, our data show that the performance of CSF T-SPOT is not as good as that of other body fluid T-SPOT, such as pleural fluid T-SPOT (64), which indicates that enough number of TB-specific lymphocytes may not always be noted in the CSF of patients with TBM. This may lead to a decrease in sensitivity for CSF T-SPOT. The possible reason for this phenomenon could be the protective effect of the blood–brain barrier. Although TB-inflamed blood–brain barrier with increased permeability allows some lymphocyte migration, the number of TB-specific lymphocytes in the subarachnoid cavity is far lower than that in the pleural cavity. Moreover, another important point that should be mentioned is that the results of CSF T-SPOT may depend on the number of CSFMCs added to the well, as the high background was reported in some studies (41, 64). As a result, it is essential to standardize technical parameters, including the number of cells, time of incubation, and the criteria of result interpretation to achieve consistent results in clinical practice. Also, QuantiFERON-TB Gold in Tube, another kind of commercially available interferon-gamma release assays, was rarely reported in diagnosing TBM. More data are needed to determine this issue in the future.

When comparing with other methods, CSF T-SPOT has no advantages over ADA in TBM diagnosis. Considering the commonly increased level of ADA in many infectious diseases, the diagnostic advantages and disadvantages of these two methods need to be further verified. On the other hand, because of lower cost and easier assessment of CSF ADA, it is undeniable that the clinical application of T-SPOT is limited. Although some studies demonstrated a relatively high sensitivity of GeneXpert MTB/RIF, it should be noticed that the use of the absolute gold standard of microbiological confirmation is likely to lead to overestimates of diagnostic sensitivity in these studies (54). In other words, GeneXpert MTB/RIF lacks enough sensitivity to distinguish TBM from non-TBM reliably and is prone to producing false-negative results because sufficient bacilli-containing CSF samples are difficult to obtain, especially from children and individuals coinfected with HIV (65, 66). Furthermore, GeneXpert MTB/RIF Ultra was reported to have higher sensitivity than GeneXpert MTB/RIF, suggesting that this new assay can provide more evidence of MTB in CSF than current bacteriological and molecular tests (67). However, given the current lack of research in this area, this new method still needs further validation. Diagnostic problems usually require holistic solutions, and further use of comprehensive criterion based on a combination of clinical and laboratory results may be indispensable to solve this problem. Also, high-quality and well-designed studies are required, as small-scale studies without validation are insufficient to establish a new diagnostic algorithm.

Our study has some limitations. First, the included studies in our meta-analysis included clinically diagnosed cases. Some diagnostic errors may have existed, which may affect the results. Second, regarding that most participants in the included studies are adult patients, the utility of T-SPOT in children with TBM is ignored, although they are the major sufferers for EPTB. Finally, there is substantial heterogeneity between the included studies. However, no potential factor was found to contribute significantly to heterogeneity on the multivariate meta-regression modeling, highlighting the need for larger, more rigorous studies.

In summary, our study suggests that both PB T-SPOT and CSF T-SPOT could severe as auxiliary tools with moderate accuracy in TBM diagnosis. Specificity is enhanced when the assay is used on CSF, but large volumes are required to get adequate CSFMCs, which hinders its clinical use.

## Data Availability Statement

All datasets presented in this study are included in the article/supplementary material.

## Author Contributions

YL and YX conceived and designed the study, analyzed the data, wrote the paper, prepared figures and tables, and reviewed drafts of the paper. XG, QL, LM, GT, and HS contributed to data analysis. FW and ZS reviewed drafts of the paper. All authors contributed to the article and approved the submitted version.

## Conflict of Interest

The authors declare that the research was conducted in the absence of any commercial or financial relationships that could be construed as a potential conflict of interest.
